# High-resolution live imaging reveals axon-glia interactions during peripheral nerve injury and repair in zebrafish

**DOI:** 10.1242/dmm.018184

**Published:** 2015-06-01

**Authors:** Yan Xiao, Adèle Faucherre, Laura Pola-Morell, John M. Heddleston, Tsung-Li Liu, Teng-Leong Chew, Fuminori Sato, Atsuko Sehara-Fujisawa, Koichi Kawakami, Hernán López-Schier

**Affiliations:** ^1^ Research Unit Sensory Biology & Organogenesis, Helmholtz Zentrum München, 85764 Munich, Germany; ^2^ Cell & Developmental Biology, Centre for Genomic Regulation, 08003 Barcelona, Spain; ^3^ Janelia Research Campus, Howard Hughes Medical Institute, Ashburn, VA 20147, USA; ^4^ Institute for Frontier Medical Sciences, Kyoto University, Shogoin, Sakyo-ku, Kyoto 606-8507, Japan; ^5^ Division of Molecular and Developmental Biology, National Institute of Genetics, The Graduate University for Advanced Studies (Sokendai), 1111 Yata, Mishima, Shizuoka 411-8540, Japan; ^6^ Department of Genetics, The Graduate University for Advanced Studies (Sokendai), 1111 Yata, Mishima, Shizuoka 411-8540, Japan

**Keywords:** High-resolution imaging, Neurotrauma, Regeneration, Schwann cells, Haptotaxis

## Abstract

Neural damage is a devastating outcome of physical trauma. The glia are one of the main effectors of neuronal repair in the nervous system, but the dynamic interactions between peripheral neurons and Schwann cells during injury and regeneration remain incompletely characterized. Here, we combine laser microsurgery, genetic analysis, high-resolution intravital imaging and lattice light-sheet microscopy to study the interaction between Schwann cells and sensory neurons in a zebrafish model of neurotrauma. We found that chronic denervation by neuronal ablation leads to Schwann-cell death, whereas acute denervation by axonal severing does not affect the overall complexity and architecture of the glia. Neuronal-circuit regeneration begins when Schwann cells extend bridging processes to close the injury gap. Regenerating axons grow faster and directionally after the physiological clearing of distal debris by the Schwann cells. This might facilitate circuit repair by ensuring that axons are guided through unoccupied spaces within bands of Büngner towards their original peripheral target. Accordingly, in the absence of Schwann cells, regenerating axons are misrouted, impairing the re-innervation of sensory organs. Our results indicate that regenerating axons use haptotaxis as a directional cue during the reconstitution of a neural circuit. These findings have implications for therapies aimed at neurorepair, which will benefit from preserving the architecture of the peripheral glia during periods of denervation.

## Abstract (precis)

**Summary:** Schwann cells are important components of the peripheral glia. We use microsurgery and high-resolution live imaging to show how Schwann cells control the regeneration of a sensorineural circuit.

## INTRODUCTION

Peripheral neuropathies in humans occur equally across ethnic, sex, age and socio-economic groups, but are more pernicious among the elderly, the diabetic and the overweight. The National Institutes of Health, for example, lists over 1500 clinical trials for neuropathies of the peripheral nervous system, demonstrating the immense interest in this global health problem (http://clinicaltrials.gov/ct2/results?term=peripheral+Neuropathy&Search=Search; accessed September 2014). Schwann cells are an important glial cell type in the vertebrate peripheral nervous system (Jessen and Mirsky, 2005; Kidd et al., 2013), and the interaction between Schwann cells and neurons is essential to maintain the integrity and function of neuronal circuits (Arthur-Farraj et al., 2012; Court et al., 2004; Nave, 2010; Rodrigues et al., 2011; Sharghi-Namini et al., 2006; Shy, 2009). Understanding neuron-glia interactions is therefore of utmost importance to devise effective treatments for peripheral neuropathies. However, a detailed characterization of the dynamic relationship between Schwann cells and neurons during injury and repair has been hampered by the size, complexity and inaccessibility of most parts of the mammalian nervous system (Erturk et al., 2012; Kang and Lichtman, 2013; Kang et al., 2014; Misgeld et al., 2007). By contrast, fish have a superficial and accessible sensory-neural system called the lateral line (Ghysen and Dambly-Chaudiere, 2007). The peripheral axons of the lateralis afferent neurons are associated with myelinating Schwann cells (Brosamle and Halpern, 2002; Lyons et al., 2005; Raphael and Talbot, 2011). Mutagenic and ultrastructural studies have revealed strong genetic and anatomical similarities between piscine and mammalian Schwann cells (Lyons et al., 2005; Pogoda et al., 2006; Czopka and Lyons, 2011; Goessling and North, 2014; Hall, 1999; Monk et al., 2009, 2013; Ruohonen et al., 2005; Sulaiman and Gordon, 2009; Sulaiman et al., 2002; Weinberg and Spencer, 1978). Accordingly, we have established an experimental paradigm that combines traumatic neural injury and multiparametric intravital microscopy in zebrafish to study in detail the behavior and function of Schwann cells during the repair of a sensory circuit.

## RESULTS

### The Tg[gSAGFF202A] transgenic line is a specific Gal4 driver in Schwann cells

**Fig. 1.**
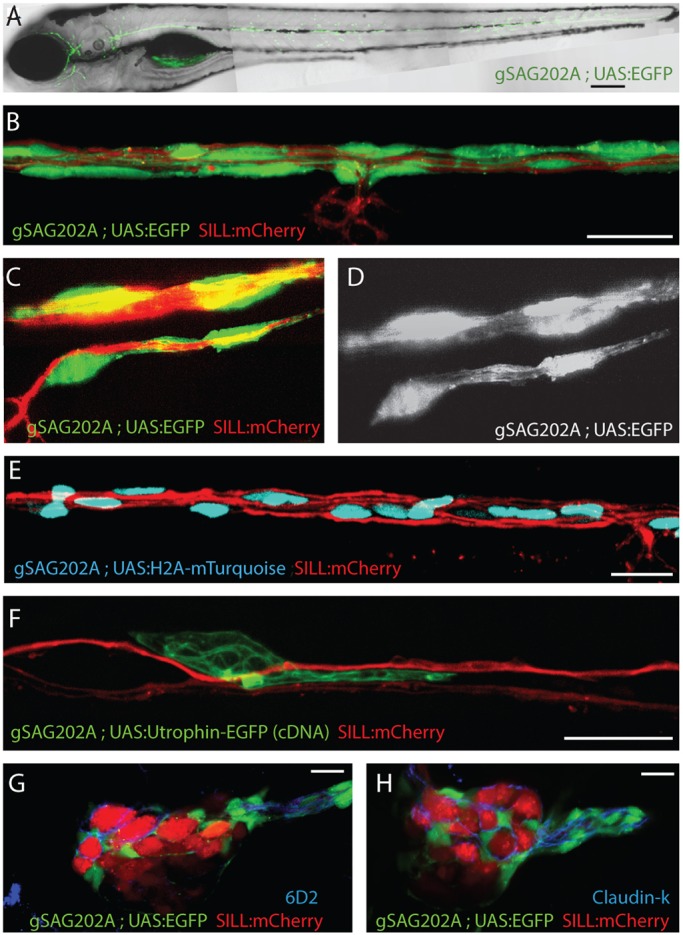
**Tg[gSAGFF202A] is a specific Gal4 driver in Schwann cells.** (A) EGFP expression pattern at 5 dpf by Tg[gSAGFF202A;UAS:EGFP]. (B-D) Triple transgenic Tg[gSAGFF202A;UAS:EGFP;SILL:mCherry] at 5 dpf show that EGFP(+) cells form tubes wrapping around an mCherry(+) axon by confocal (B) and lattice light-sheet (C,D) imaging. (E) A triple-transgenic larva (Tg[gSAGFF202A;UAS:H2A- mTurquoise;SILL:mCherry]) at 5 dpf. Nuclei of cells that are in intimate contact with lateralis afferent axons are shown in blue along the trunk. (F) Marking of individual cell shows UtrCH-EGFP expression in a 4-dpf Tg[gSAGFF202A;SILL:mCherry] double-transgenic fish injected with UAS:UtrCH-EGFP construct. (G) The marker 6D2 labels EGFP-expressing cells in a lateral plane of posterior ganglion. (H) The marker Claudin-k labels EGFP-expressing cells in a lateral plane of posterior ganglion. In all figures, dorsal is up and anterior is left. Scale bars: 150 μm (A) and 10 μm (B-H).

Transgenic zebrafish lines expressing fluorescent proteins have been valuable tools to study Schwann-cell behavior during embryogenesis (Jung et al., 2010; Lewis and Kucenas, 2013; Munzel et al., 2012; Perlin et al., 2011). However, additional resources are needed to visualize and manipulate Schwann cells in the intact animal. In the zebrafish and other species, cell-type-specific ‘driver’ transgenic lines that produce the transcription factor Gal4 can express ‘effector’ genes under the control of an ‘upstream activating sequence’ (UAS) (Asakawa and Kawakami, 2008; Faucherre and López-Schier, 2011; Halpern et al., 2008; Scheer and Campos-Ortega, 1999; Scott et al., 2007). To our knowledge, no Schwann-cell-specific driver line exists in zebrafish. To overcome this deficiency, we screened a large collection of transgenic strains that were generated by random integration of a gene-trapping construct bearing a Gal4FF driver (Kawakami et al., 2010). We obtained one line called Tg[gSAGFF202A] that activated the expression of UAS-controlled green-fluorescent protein (EGFP) in cells along the anterior and posterior lateral line in larval stages (Fig. 1A). It was not initially possible to unambiguously identify the cells marked by Tg[gSAGFF202A;UAS:EGFP] because lateralis afferent axons and Schwann cells are in intimate contact. Upon closer examination, we found that neuronal central projections were not evident in the head of these transgenics (Fig. 1A), suggesting that Gal4 expression was not in afferent or efferent neurons. To further test this assumption, we crossed Tg[gSAGFF202A;UAS:EGFP] with the Tg[SILL:mCherry] line, which expresses mCherry in afferent neurons (Pujol-Martí et al., 2012). Examination of the resulting triple-transgenic larvae by laser-scanning confocal microscopy revealed that EGFP and mCherry were not co-expressed (Fig. 1B). In particular, EGFP was not present in the terminal arborization of the afferent axons below the neuromasts (Fig. 1B). To visualize the EGFP-expressing cells at higher resolution, we used a novel imaging technology called lattice light-sheet microscopy, which is based on applying a thin sheet of patterned light onto living specimens to reveal sub- and supra-cellular structures in three dimensions at high speed (Chen et al., 2014). Lattice light-sheet imaging showed that the EGFP(+) cells formed tubes containing the mCherry(+) axons, revealing that the Gal4-expressing cells were perineural (Fig. 1C,D and supplementary material Movie 1). To better characterize these cells we generated a new stable transgenic line expressing the blue-fluorescent protein mTurquoise fused to Histone-2A under the control of UAS (Goedhart et al., 2010), which targets mTurquoise to the nucleus of Gal4-expressing cells. Tg[gSAGFF202A;UAS:H2A-mTurquoise;SILL:mCherry] triple transgenics showed blue-fluorescent nuclei in cells along the entire lateral line. Importantly, lateralis afferent axons were not labeled by mTurquoise (Fig. 1E). We also expressed the F-actin-binding domain of the calponin-homology domain of Utrophin (Utr-CH) fused to EGFP (Wuhr et al., 2011) in single Gal4(+) cells in Tg[gSAGFF202A;SILL:mCherry]. This showed discrete cells in intimate association with lateralis axons (Fig. 1F). To further characterize these cells, we labeled transgenic larvae with the monoclonal antibody 6D2, which recognizes a carbohydrate epitope of piscine P0-like myelin glycoprotein in peripheral glial cells (Bastmeyer et al., 1994; López-Schier and Hudspeth, 2005). This antibody marked the EGFP-expressing cells but not lateralis neuronal perykaria (Fig. 1G). To further determine their identity, we used an antibody to the Claudin-k junctional protein, which labels Schwann cells in peripheral neurons of zebrafish (Munzel et al., 2012). This antibody decorated all the Gal4(+) cells (Fig. 1H). These data demonstrate that the Tg[gSAGFF202A] transgenic line expresses Gal4 in Schwann cells.

**TRANSLATIONAL IMPACT**

**Clinical issue**

Peripheral neuropathies are nerve pathologies caused by genetic disorders or physical trauma, which in severe cases can cause transient sensory and motor dysfunction. Experimental and clinical evidence indicates that defective neural regeneration is associated with definitive loss of sensory-motor capacity or pain. Notwithstanding recent progress, our knowledge about the interaction between injured and regenerating neurons and glial cells remains incomplete to the point that therapies against neural injury are often ineffective. Differential cell vulnerability to denervation further complicates efforts to elucidate the basis of degeneration and regeneration.

**Results**

In this study, the authors established an experimental model of neurotrauma to study the fundamental mechanisms that underlie neuronal and glial interactions *in vivo* at high spatiotemporal resolution. They developed an *in vivo* assay using the zebrafish, an animal model that combines the presence of long-fiber sensory neurons and their associated glia (Schwann cells) with the availability of both supra-cellular high-resolution live imaging and genetic and microsurgical manipulations. The authors conducted a comprehensive characterization of Schwann cells and neurons during homeostasis, physical injury and repair by intravital imaging using standard and novel imaging techniques. Results show that denervation induces progressive loss of Schwann cells by inducing their apoptosis. In addition, they show that the negative effects of denervation are reversible because Schwann-cell re-innervation prevents further glial destruction.

**Implications and future directions**

Dynamic processes in the nervous system should be studied *in toto* because the cells in their natural context provide the ideal framework for evaluating changes associated with physical injury. The zebrafish has a similar and simpler version of the mammalian peripheral nervous system that is amenable to high-resolution intravital imaging. This is very important for direct observation of the organs, which is very challenging in mammals. The data obtained from this study provide basic mechanistic insights about the onset and progression of traumatic neuropathies, and could help delineate the genetic networks underlying neurodegeneration and neurorepair. These results have direct clinical implications because they demonstrate the dominant influence of the Schwann cells on the onset and directionality of axonal regeneration after injury. Thus, strategies aimed at neural-circuit repair might benefit from preventing the disassembly of the glia during periods of denervation. Therefore, a future translational outcome of this study is the identification of drugs that can maintain the integrity of the glia to treat traumatic neuropathies in humans.

### The Tg[gSAGFF202A] insertion disrupts the *ErbB2* gene

**Fig. 2.**
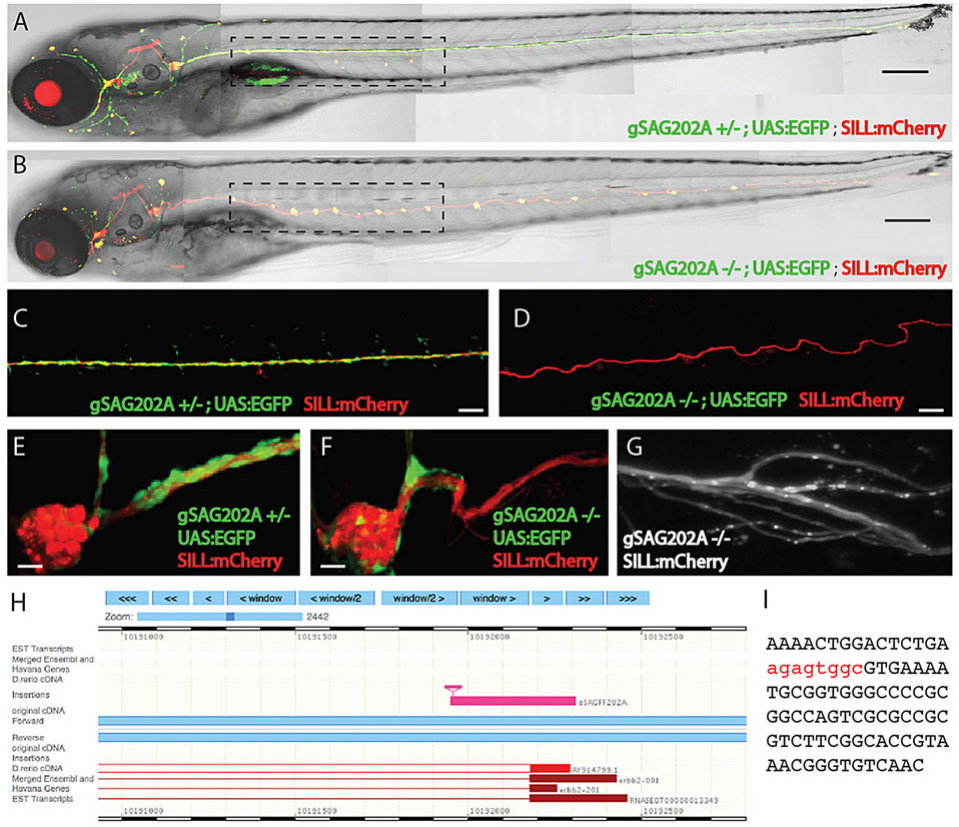
**The phenotype of homozygous Tg[gSAGFF202A] fish.** (A) Heterozygous Tg[gSAGFF202A;UAS:EGFP;SILL:mCherry] 5-dpf larva treated with 4-Di-2-ASP, revealing the normal pattern of neuromasts in its posterior lateral line (five neuromasts within the dashed rectangle). (B) A homozygous Tg[gSAGFF202A;UAS:EGFP;SILL:mCherry] fish at 5 dpf possesses around twice as many neuromasts (ten neuromasts within the dashed rectangle). (C,E) The normal expression pattern of the posterior ganglion and lateral line in heterozygous Tg[gSAGFF202A;UAS:EGFP;SILL:mCherry] fish at 5 dpf. (D,F) At 5 dpf, the abnormal expression pattern of the posterior ganglion and lateral line in homozygous Tg[gSAGFF202A;UAS:EGFP;SILL:mCherry] fish, in which Schwann cells cannot migrate far away from the posterior ganglion. (G) Lattice light-sheet imaging reveals details of the axonal defasciculation in homozygous Tg[gSAGFF202A;UAS:EGFP;SILL:mCherry] larvae at 5 dpf. (H) BLAST (basic local alignment search tool) shows the insertion of Gal4FF within the *ErbB2* gene. (I) The integration site indicated by red nucleotides. Scale bars: 150 μm (A,B), 50 μm (C,D) and 10 μm (E-G).

One quarter of the progeny from crossings of Tg[gSAGFF202A] transgenic males and females presented supernumerary neuromasts, suggesting that the insertion is mutagenic (Fig. 2A-F and supplementary material Fig. S1). Zebrafish lacking Schwann cells show this phenotype, in addition to nerve defasciculation due to loss of myelination. Using confocal and lattice light-sheet microscopy, we also observed fasciculation defects in Tg[gSAGFF202A] homozygous larvae (Fig. 2C,D,G). Therefore, we positionally mapped the Tg[gSAGFF202A] transgene and found that it is inserted in the first coding exon of the *ErbB2* locus (Fig. 2H,I). The ErbB2 receptor tyrosine kinase has been shown to be essential for the migration of Schwann cells along growing lateralis afferent axons in zebrafish (Grant et al., 2005; López-Schier and Hudspeth, 2005; Lush and Piotrowski, 2014; Lyons et al., 2005). Genetic crossings showed that the *ErbB2^st61^* mutant allele did not complement Tg[gSAGFF202A] (data not shown) (Lyons et al., 2005). Thus, the Tg[gSAGFF202A] insertion represents a new recessive, fully penetrant and strongly expressive loss-of-function allele of *ErbB2*.

### Differential responses of Schwann cells to acute and chronic denervation

**Fig. 3.**
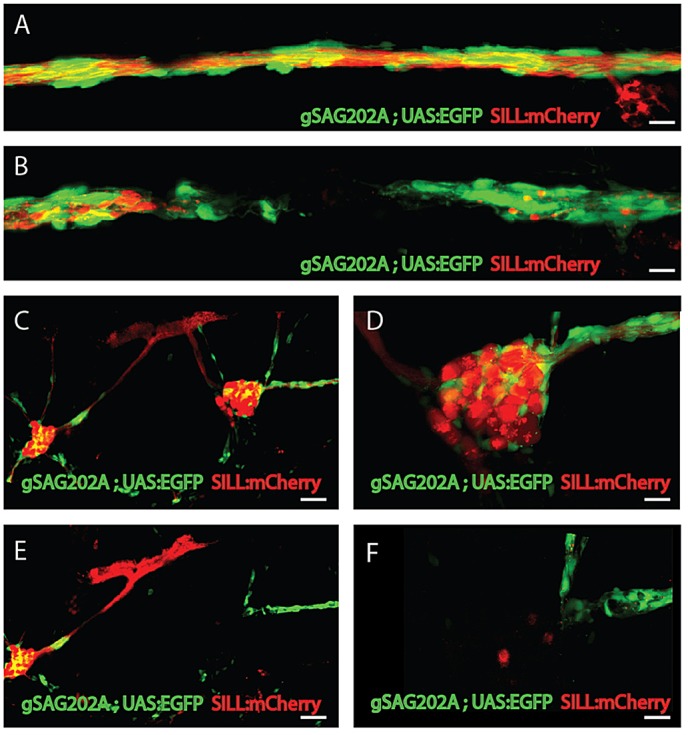
**Selective axonal severing and neuronal ablation by laser>.** (A,B) High magnification of the lateral line nerve (A) before laser ablation and (B) after laser ablation. The nerve and Schwann cells are precisely and completely severed with no visible damage to the surrounding tissue. (C) The maximal projections of anterior and posterior ganglion before ablation. (D) Higher magnification of posterior ganglion before ablation. (E) The image of the same animal after ablation reveals that the posterior ganglion was totally ablated. (F) Higher magnification of posterior ganglion after ablation. Scale bars: 10 μm (A,B,D,F) and 50 μm (C,E).

**Fig. 4.**
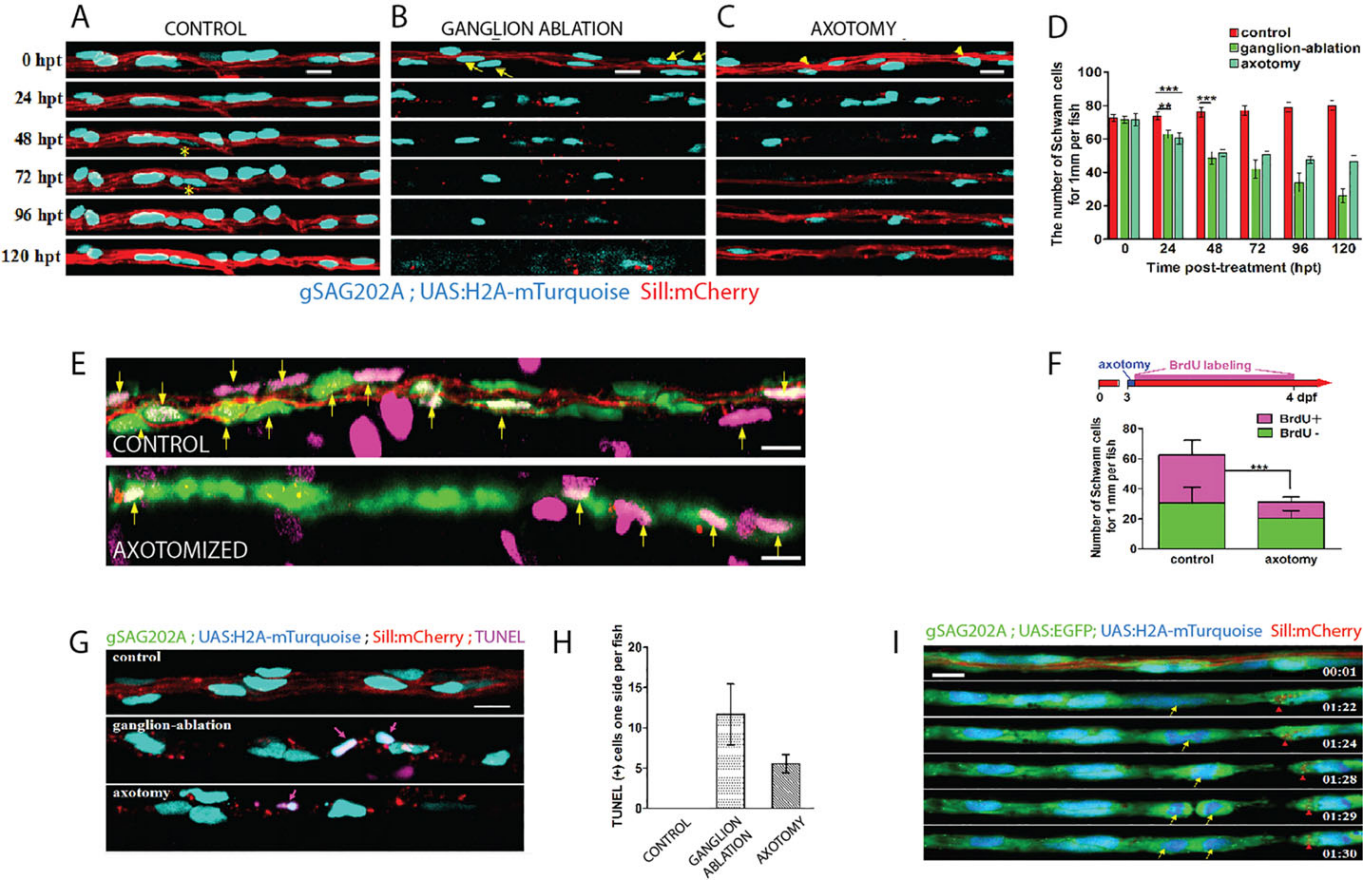
**Differential responses of Schwann cells to acute and chronic denervation.** (A-C) Tg[gSAGFF202A;UAS:H2A-mTurquoise;SILL:mCherry] larvae, starting at 5 dpf and following 24 hpt, 48 hpt, 72 hpt, 96 hpt and 120 hpt in the control (A), ganglion-ablated (B) and axon cuts (C) groups. Yellow asterisks mark the proliferative cells. Yellow arrows and arrowheads indicate the dead cells. (D) Quantification of Schwann cells, showing a significant reduction in the group with severed axons (*n*=6) and the ganglion-ablated group (*n*=8) compared with the control group (*n*=5) (**P<0.001, ***P<0.0001). (E) Control and axon-transected Tg[gSAGFF202A;UAS:EGFP;SILL:mCherry] larvae treated with BrdU. (F) The larvae were subject to axon severing at 3 dpf in 10 mM BrdU until 24 hpt followed by fresh embryo medium. Fewer BrdU-positive Schwann cells were observed in the axon-transected (*n*=8) compared with the control (*n*=8) group (***P<0.0001, two-tailed *t*-test). (G) Examination of apoptotic Schwann cells by TUNEL assay. Pink arrows indicate colocalization of H2A-mTurquoise with TUNEL-positive cells. (H) Quantification of TUNEL-positive Schwann cells in the control group, the ganglion-ablation group and the axon-severed group. (I) Frames captured from supplementary material Movie 2 of a Tg[gSAGFF202A;UAS:EGFP;UAS:H2A-mTurquoise;SILL:mCherry] larva with severed axons. Numbers in the lower right corners denote time elapsed from the first frame of the movie. Red arrowheads mark axon debris that has been phagocytosed by the Schwann cells. At 10 hpt (01:24 time point), Schwann cells started to divide (yellow arrow) when no intact axon exists. Error bars are+or±s.d. Scale bars: 10 μm (A-C,E,G,I).

The peripheral axons of zebrafish sensory neurons, including those of the lateral line, are able to quickly regenerate after injury (Graciarena et al., 2014; Villegas et al., 2012; Martin et al., 2010). Neuronal ablation, by contrast, abolishes lateral-line innervation for a longer period. Here, we designed a method to generate acute or chronic denervation of Schwann cells in whole animals, by axon severing (Fig. 3A,B) or neuronal ablation (Fig. 3C-F), respectively. The strategy consists of inducing plasma-mediated cell damage by directing a pulsed ultraviolet laser (355 nm, 400 ps/2.5 μJ per pulse) onto the living specimen. The laser was coupled to a spinning-disc inverted microscope and focused to axons for severing (Fig. 3A) or neuronal soma for ablation (Fig. 3C,D) in Tg[SILL:mCherry] transgenics. Observation at 1 h after microsurgery confirmed complete severing (Fig. 3B) or neuronal ablation (Fig. 3E,F). Next, we used Tg[gSAGFF202A;UAS:H2A-mTurquoise;SILL:mCherry] triple transgenics starting at 5 dpf (days post-fertilization) to quantify Schwann cells after denervation (Fig. 4A-D). A follow-up period of 5 days showed a significant decrease in the number of Schwann cells marked with H2A-mTurquoise beginning at 24 h post-trauma (hpt) (Fig. 4B-D). Schwann-cell loss continued in the fish lacking afferent neurons but ceased in fish with transected axons at 48 hpt (Fig. 4A-D). We reasoned that the decrease of Schwann cells upon denervation might be due to the loss of fluorescent-protein expression in this particular transgenic line. However, we did not favor this possibility for two reasons. First, we used H2A-mTurquoise as a Schwann-cell marker. Although we have not directly measured the stability of the H2A-mTurquoise fusion protein, histones are known to be some of the most stable proteins in animal cells, with a half-life of several months (Toyama et al., 2013), suggesting that even if Schwann cells ceased to express Gal4 immediately after denervation, fluorescence would persist in nuclei for longer than our 5-day follow-up period. Second, we did not observe widespread loss of H2A-mTurquoise, which would be expected if Schwann cells ceased to express Gal4 after denervation (Fig. 4B-D). Alternatively, glial depletion upon denervation might result from increased Schwann-cell apoptosis, or by physiological cell death accompanied by abnormally reduced Schwann-cell proliferation. To test these possibilities we performed a 24-h pulse-chase analysis of DNA synthesis by bromodeoxyuridine (BrdU) incorporation in Schwann cells as a proxy for cellular proliferation, starting at 3 dpf (Fig. 4E,F). We quantified Schwann cells within 1 mm of the fish trunk and found that approximately 50% of the Schwann cells were BrdU(+) in control animals [BrdU(+) 32.1±5.9 cells; BrdU(−) 31.4±8.2 cells; *n*=8], suggesting that these cells actively proliferate (Fig. 4F). Upon acute denervation, we observed the expected reduction in Schwann-cell content, and that only around 33.6% of the glia were BrdU(+) [BrdU(+) 10.5±4.5 cells; BrdU(−) 20.8±8.5 cells; *n*=8 larvae] (Fig. 4F). To assess cell death, we used the TUNEL assay in Tg[gSAGFF202A;UAS:H2A-mTurquoise;SILL:mCherry] transgenics starting 24 h after denervation. TUNEL-positive staining in Schwann cells was rarely observed in the control fish (*n*=5), whereas prominent and widespread TUNEL signal colocalized with H2A-mTurquoise in the experimental specimens [TUNEL(+) Schwann cells 11.7±3.8 in larvae with ablated ganglia, *n*=8; TUNEL(+) Schwann cells 5.6±1.1 in larvae with severed axons, *n*=8], suggesting that the reduction in the number of Schwann cells resulted from cell death (Fig. 4G,H). We also directly visualized Schwann-cell dynamics by live-microscopy imaging of in the quadruple transgenics, Tg[gSAGFF202A;UAS:EGFP;UAS:H2A-mTurquoise;SILL:mCherry], which clearly showed the mitotic behavior and death by apoptosis of denervated Schwann cells (Fig. 4I and supplementary material Movie 2). We did not observe extrusion of Schwann cells from the glial stream (supplementary material Movie 2). Thus, these results indicate that denervation induces Schwann-cell death, and that the regrowth of axons prevents further glial depletion.

**Fig. 5.**
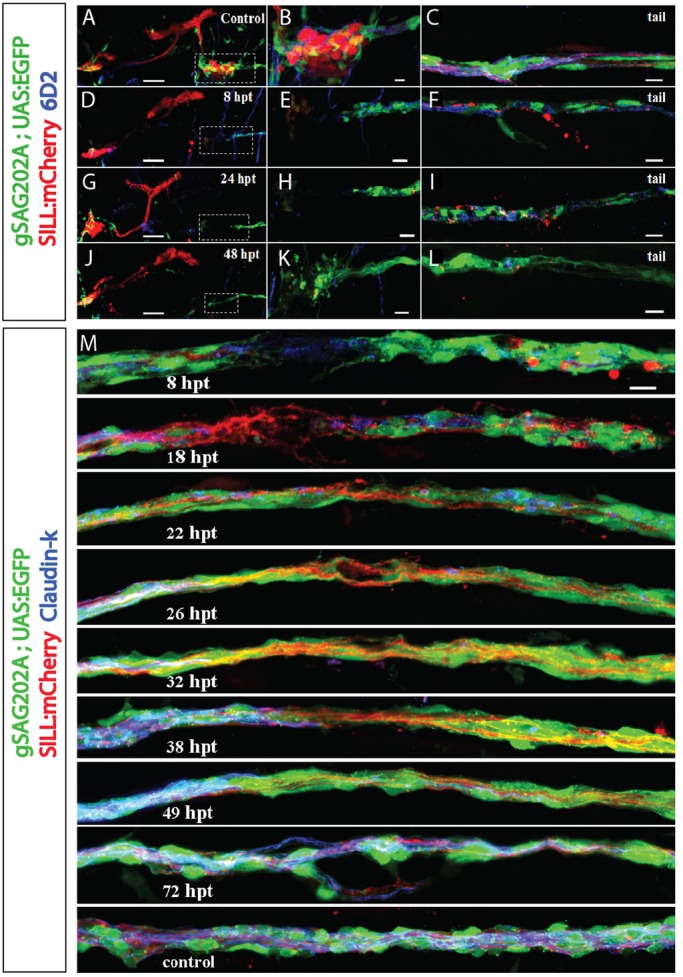
**Gradual loss of expression of myelin glycoprotein and Claudin-k junctional protein upon axon severing or ganglion ablation.** (A-L) Maximal projection of Tg[gSAGFF202A;UAS:EGFP;SILL:mCherry] larvae immunolabeled with myelin 6D2 antibody (blue). (A,D,G,J) The cephalic region showing the anterior and posterior ganglion before ganglionostomy (A) and at 8-48 hpt in fish with ganglion ablation (D,G,J). (B,E,H,K) Higher magnification of the boxed regions in A,D,G,J, respectively. (C,F,I,L) Images of the animals’ tail, corresponding to A,D,G,J. (D,G,J) At 8 hpt, 24 hpt, 48 hpt, immunostaining images reveals gradual loss of myelin protein. (F,I,L) Immunostaining of fish tails delineates the loss of myelin and clearance of axon debris in a gradual manner. (M) Time-course analysis of axonal regeneration and Claudin-k junctional protein expression (blue) in the Schwann cells around the site of damage. Claudin-k is present along the perineurium in control fish (lowest panel). A marked reduction of Claudin-k in the perineurium distal to the cutting site can be seen at 8 hpt. Distal Claudin-k protein levels remain lower than on the proximal part of the perineurium even after axonal regeneration at 26 hpt, and only level-up around 72 hpt. Scale bars: 50 μm (A,D,G,J), 10 μm (all others).

To assess architectural changes of denervated peripheral glia, we used the 6D2 and the anti-Claudin-k antibodies (Fig. 5A-L). In control animals, 6D2 decorated Schwann cells around the lateralis afferent ganglia and neuronal peripheral axons (Fig. 5A-C). Upon laser ablation of the posterior ganglion (Fig. 5D), the central axons and projections rapidly degraded. Schwann cells around the ganglion were also ablated with the laser (Fig. 5D,E). We chose samples that presented no newborn neurons within the period of analysis (Fig. 5D,G,J). At 24 hpt, Schwann cells devoid of axons began to lose expression of the P0-like myelin glycoprotein (Fig. 5G-I), and had lost virtually all 6D2 labeling at 48 hpt (Fig. 5J-L). A detailed visualization of the trunk of the treated specimens showed Claudin-k uniformly distributed along the lateralis nerve (Fig. 5M). After axonal severing, the Schwann cells around the proximal nerve stump maintained Claudin-k protein, whereas the Schwann cells in contact with the distal degenerating axons began to lose Claudin-k protein expression starting at 8 hpt (Fig. 5M). The levels of Claudin-k distal to the site of injury remained lower than on the proximal part, even after axonal regeneration at 26 hpt (Fig. 5M). However, Claudin-k protein levels in Schwann cells returned to normal and were equivalent on both sides of the axonal cuts around 72 hpt (Fig. 5M).

### Schwann cells facilitate but are dispensable for axonal regrowth

**Fig. 6.**
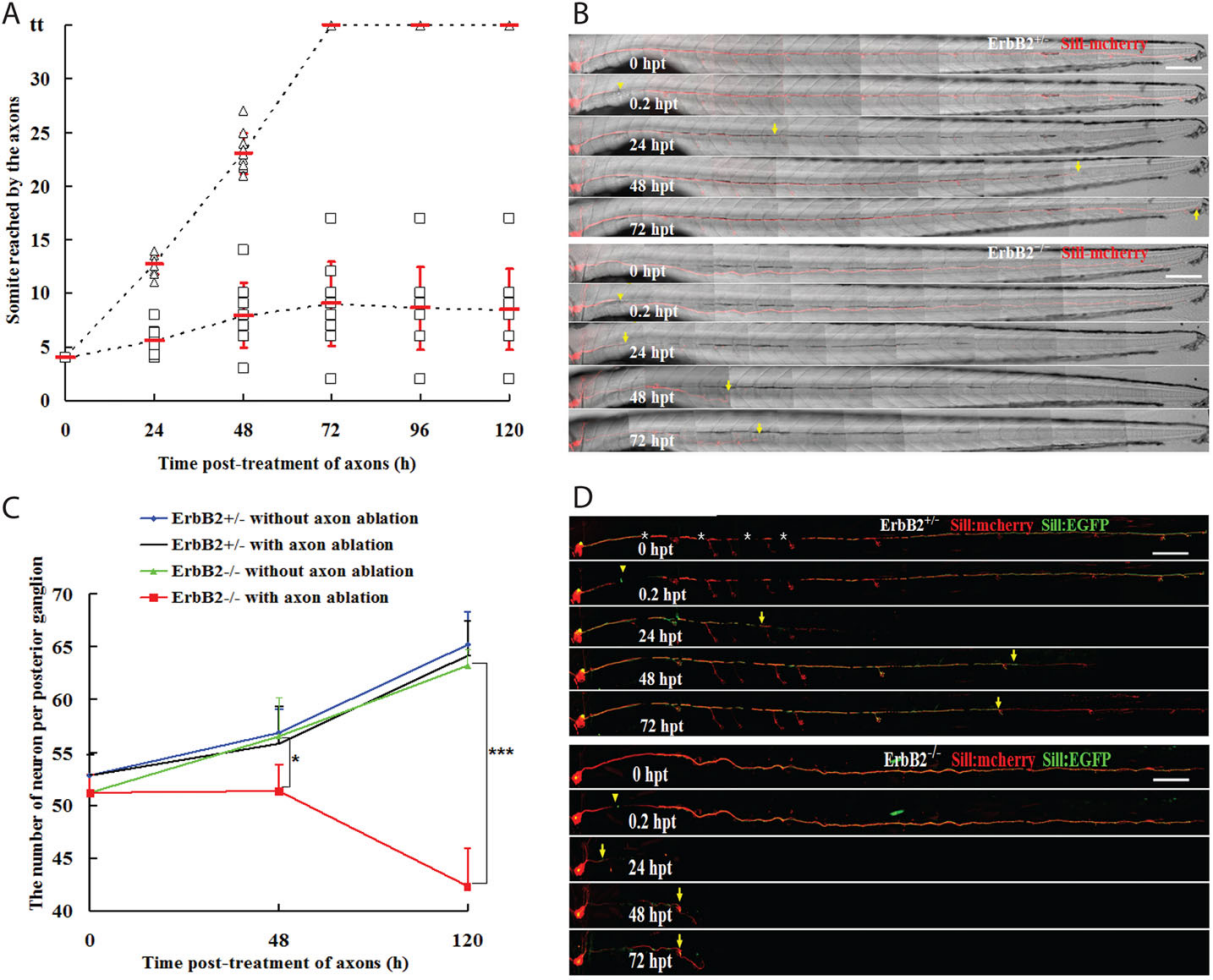
**Schwann cells facilitate but are dispensable for axonal regeneration.** (A) The axons were severed at somite 3 in 5-dpf larvae. The somite reached by the regenerating nerve was examined at 24, 48, 72, 96 and 120 hpt. In all cases (*n*=10), the axons had reached the tip of the body and re-innervated the terminal neuromasts at 72 hpt in Tg[gSAGFF202A+/−;SILL:mCherry] (triangles). All cases (*n*=10) of Tg[gSAGFF202A−/−;SILL:mCherry] fish revealed an abnormal and lower regeneration rate (squares). (B) Examples of axon regeneration after injury in heterozygous and homozygous Tg[gSAGFF202A;SILL:mCherry]. (C) Sum of neuron numbers in posterior ganglion at 5 dpf, 48 hpt and 120 hpt in Tg[gSAGFF202A+/−;SILL:mCherry] and Tg[gSAGFF202A−/−;SILL:mCherry] larvae upon laser-mediated axon severing or in the untreated group. *n*=6 fish per group (*P<0.05, ***P<0.0001). (D) Examples of an identified axon’s regeneration after damage in heterozygous and homozygous Tg[gSAGFF202A;SILL:mCherry] fish injected with SILL:EGFP. Yellow arrowheads and arrows point to cutting sites and to the tip of regenerate axons, respectively. White asterisks mark pigment cells. Error bars are+or±s.d. Scale bars: 150 μm (B,D).

To investigate the influence of Schwann cells on the rate and extent of axon regrowth after injury, we used the double-transgenic larvae Tg[gSAGFF202A;SILL:mCherry] at 5 dpf (Fig. 6A), when the posterior lateralis ganglion contains around 50 neuronal perikarya. The stereotypic position of this ganglion and the lateral branches of the afferent axons towards the neuromasts allowed us to precisely define a site of axonal cuts between samples (Fig. 6B). Additionally, we used the somites along the trunk to quantify the extent of axonal regeneration (Fig. 6B). All afferent axons were severed between the posterior ganglion and the first neuromast situated between somites 5 to 7. A time-course analysis of axonal regeneration in the double-transgenic Tg[gSAGFF202A+/−;SILL:mCherry] fish that we used as wild-type controls revealed that severed axons began to regrow within 24 hpt. On average, the fastest growth cones reached 12.7±0.9 somites at 24 hpt, and continued to grow at an average speed of 0.4±0.1 somites per hour until they reached the tip of the tail, which occurred within 72 hpt (Fig. 6A,B). By contrast, in Tg[gSAGFF202A−/−] mutants, axons showed a much slower rate of regrowth, advancing on average to somite 5 by 24 hpt, and stabilizing at around somite 9 by 72 hpt, with negligible further growth and often some retractions during the remainder of the follow-up period (Fig. 6A,B).

The defective circuit recovery in the *ErbB2* mutants might be due to neuronal death. Therefore, we quantified the neuronal population in wild-type and *ErbB2* mutant fish under control and traumatic conditions. We counted perikarya using confocal stacks of the posterior ganglion in Tg[gSAGFF202A;SILL:mCherry]. Wild-type larvae at 5 dpf contained around 53 neurons, whereas the average number of neurons in *ErbB2* mutants was 51 (Fig. 6C). Over the course of 5 days, ganglia grew to 65.2±3.1 and 63.2±1.7 neurons by 10 dpf in wild-type and *ErbB2* mutants, respectively (*P*=0.097). Axon severing did not affect neuronal number or ganglion growth in wild-type animals (48 hpt: control 56.8±2.2 neurons, severed axons 55.8±3.6 neurons, *P*=0.81; 120 hpt: control 65.2±3.1 neurons, severed axons 64.2±3.3 neurons, *P*=0.65). However, axon severing had a profound effect on neuronal viability in the *ErbB2* mutants (48 hpt: control 56.5±3.7 neurons, severed axons 51.3±2.5 neurons, *P*<0.05; 120 hpt: control 63.2±1.7 neurons, severed axons 42.3±3.6 neurons, *P*<0.0001) (Fig. 6C). Neural-circuit recovery in wild-type fish might result either from the efficient regrowth of damaged axons, from the growth of axons of newborn neurons (∼two new neurons per day) or from a combination of the two processes. Whole-fascicle severing experiments did not allow the discrimination between axonal regrowth and axonogenesis by newborn neurons. To directly test the growth capacity of severed axons in control and *ErbB2* mutants, we severed the peripheral axon of a single identified afferent neuron. To this end, we marked individual neurons by stochastic expression of EGFP under the transcriptional control of the SILL enhancer by DNA injections. We used the transgenic line Tg[gSAGFF202A;SILL:mCherry] to identify *ErbB2* mutants and to ensure that EGFP expressors were lateralis afferent neurons. Next, we severed these single EGFP-marked axons and found that they efficiently regrew in wild-type larvae, but not in *ErbB2* mutants (Fig. 6D). Thus, fish lacking ErbB2 activity experience elevated neuronal death and a failure to regrow axons effectively after severing. Together, these results suggest that Schwann cells play an important role during regenerative axonal growth.

**Fig. 7.**
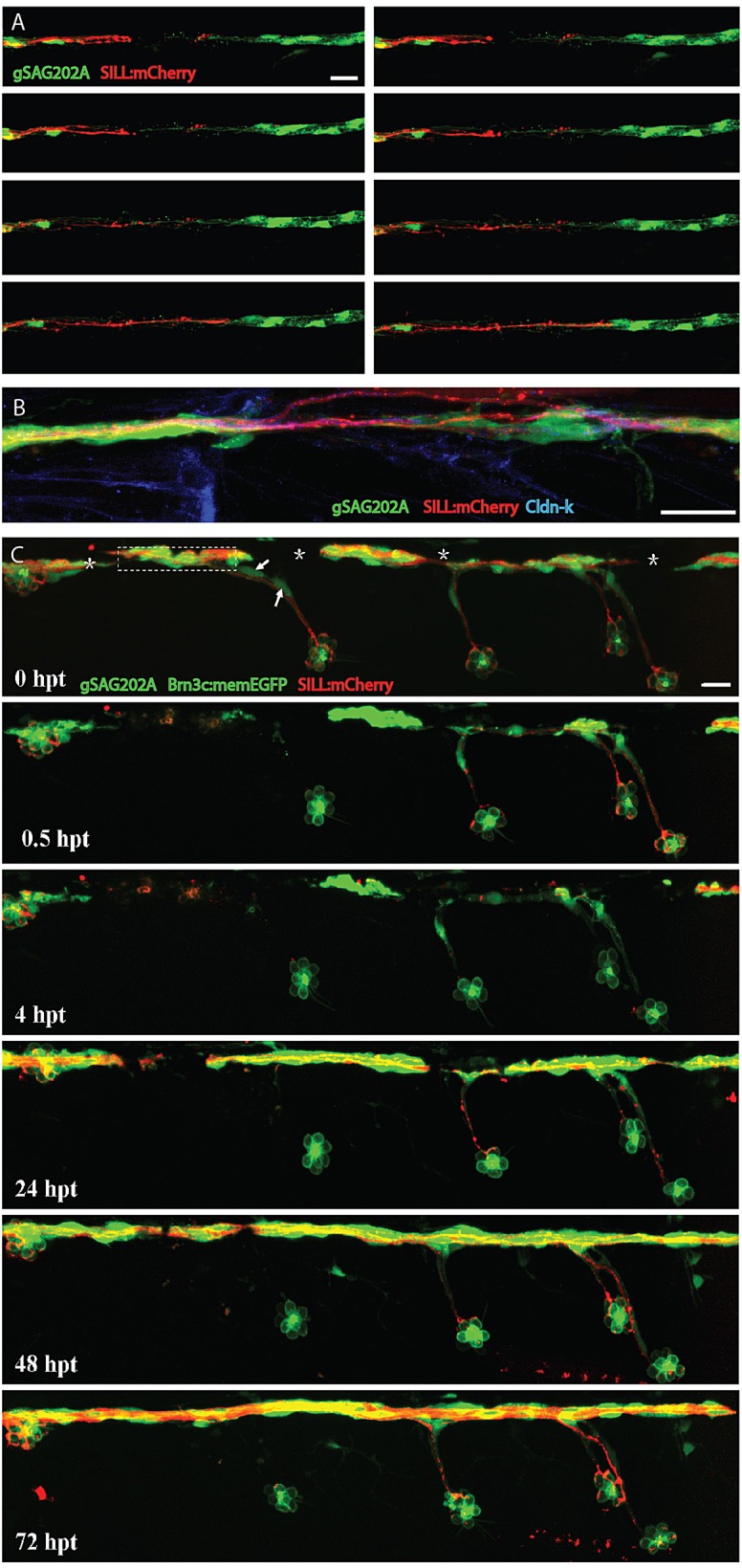
**Regenerating axons followed Schwann cell processes.** (A) Frames captured from time-lapse supplementary material Movie 4 of a Tg[gSAGFF202A;UAS:EGFP;SILL:mCherry] larva with severed axons, leaving a glial gap. Time elapsed from the first frame of the movie are, from left to right and top to bottom: 00:04, 00:20, 00:30, 00:40, 00:45, 00:46, 00:49, 00:52. Pictures were taken every 8 min, starting 4 h after axon severing. At 16 hpt (00:45 time point), axons started to regrow when Schwann cells filled the gap. (B) An example of partially defasciculated axons at the site of injury. (C) A quadruple transgenic Tg[gSAGFF202A;UAS:EGFP;SILL:mCherry;Brn3c:mEGFP] larva starting at 6 dpf and following 0.5 hpt, 4 hpt, 24 hpt, 48 hpt and 72 hpt after axon severing and ablation of lateral Schwann cells. At 24 hpt, regenerating axons re-innervated the neuromast with lateral Schwann cells, but failed to re-innervate neuromasts devoid of lateral Schwann cells even at 72 hpt. Asterisks mark pigment cells. Arrows point to ablated lateral Schwann cells. Dash frame indicates the site of the cut. Scale bars: 25 μm.

The afferent neurons of the lateral line extend pioneer axons independently of Schwann cells during embryonic development (Gilmour et al., 2002), suggesting differences between developmental axonogenesis and axonal regrowth. To assess the dynamic relationship between axons and Schwann cells during axonal regrowth we performed live imaging in Tg[gSAGFF202A;UAS:EGFP;SILL:mCherry] triple transgenics. Laser-mediated axon severing also ablated Schwann cells locally, leaving a glial gap of between 20 and 50 μm (Fig. 7A). We found that, immediately after injury, there was a retraction of the proximal and distal axon fragments, accompanied by a rapid physiological clearing of distal axonal debris by the Schwann cells (Fig. 7A and supplementary material Movies 2 and 3). Distal and proximal Schwann cells adjacent to the injury extended bridging processes towards the glial gap (supplementary material Movie 3). Axonal growth cones seemed to accompany proximal glial processes, with growth and retraction phases that alternately leave Schwann-cell processes leading and lagging the growth-cone tips (supplementary material Movie 4). High-resolution visualization of individual Schwann cells showed that they are structurally stable in unperturbed conditions (supplementary material Movie 5). However, upon axon severing, they readily extended actin-rich processes (supplementary material Movie 6). We did not observe any difference in the dynamics of extensions between proximal and distal Schwann cells (supplementary material Movie 3). Regenerating axons were found to preferentially extend growth cones around the Schwann cells and crossed the injury site as soon as the glial gap was bridged (Fig. 7A and supplementary material Movies 3 and 6). In instances when the bridging of the gap by Schwann cells was delayed, regrowing axons stalled, and only resumed growth upon physical contact with the distal Schwann cells (supplementary material Movie 4). Most regenerating axons were readily myelinated by the distal Schwann cells, but often remained defasciculated over the injury site (Fig. 7B). These results show that Schwann cells are not essential but facilitate axonal regrowth by enhancing the capacity of growth cones to negotiate the site of injury.

### Schwann cells are necessary for the re-innervation of peripheral targets

We observed that one to four ‘lateral’ Schwann cells associated with axon terminal projections below the neuromasts (Fig. 1C,D). Therefore, we used these projections to investigate whether Schwann cells play a role in the re-innervation of sensory organs by regenerating axons. To this end, we severed axons before two adjacent neuromasts (control and experimental) and killed the lateral Schwann cells of the more rostral neuromast (experimental). The Tg[gSAGFF202A;UAS:EGFP;SILL:mCherry;Brn3c:mEGFP] specimens expressing green fluorescence in hair cells enabled the identification of the experimental and the control neuromasts before and after the damage (Fig. 7C) (Faucherre et al., 2009; Xiao et al., 2005). A follow-up period of 72 h showed that Schwann cells at the main axonal bundle did not migrate to replace the ablated lateral Schwann cells, leaving the experimental neuromasts devoid of glia. Regenerating axons always re-innervated the control neuromasts but, in seven out of nine cases, failed to re-innervate the experimental neuromasts devoid of Schwann cells (Fig. 7C). This result indicates that Schwann cells serve as a cue and substratum for the growth cone of regenerating axons, and that the glia are essential for the regenerative innervation of peripheral sensory organs.

## DISCUSSION

### The experimental animal model of disease

Neuropathies arising from neuronal or glial damage due to cuts, burns and other traumatic events cause severe morbidity and increased mortality in humans (Beirowski, 2013; Scherer and Wrabetz, 2008). Understanding the cellular and molecular mechanisms involved in the onset and progression of neuropathies of glial origin is essential to develop novel therapeutic interventions (Gordon et al., 2003). Several studies have firmly demonstrated the strong conservation of neuron-glia interactions among vertebrates. However, most of the classical experimental animal models are not amenable to explore the fine details of the dynamic interaction between Schwann cells and neurons because of the inaccessibility of these cells to direct live imaging at high resolution. The zebrafish has emerged as a favorable system to study organ development, homeostasis and regeneration in the whole animal at high resolution (Goessling and North, 2014; Patton et al., 2014). Here, we use the peripheral nervous system of the zebrafish larva to establish an experimental paradigm of traumatic injury. We combine high-resolution live imaging with genetic and microsurgical manipulations to study the relationship between Schwann cells and their associated sensory neurons during injury and repair. We also implemented for the first time lattice light-sheet microscopy in zebrafish larvae. This novel imaging technology employs an ultra-thin laser sheet generated by two-dimensional optical lattices, which enabled us to perform three-dimensional intravital imaging at very high resolution (Chen et al., 2014). We predict that the combination of genetically encoded fluorescent biosensors and lattice light-sheet microscopy should further enable the visualization of subcellular physiological responses, and cytoskeletal and organellar dynamics, with unprecedented resolution. Additionally, although we have focused on axonal and circuit repair, our disease model can be easily adapted to visualize and probe other injury responses, such as inflammation, infection, immune reactions and scarring. We discuss below our results and their clinical implications.

### How do Schwann cells respond to denervation?

We found that, during the initial loss of contact with the axolemma, Schwann cells downregulate the expression of the P0-like myelin glycoprotein and junctional Claudin-k, suggesting that they de-differentiate (Scherer, 1997). Thus, Schwann cells in zebrafish and in mammals behave similarly upon denervation (Harrisingh et al., 2004). We also found that chronic denervation is lethal for Schwann cells. Although glial depletion also occurred during acute denervation, axonal regrowth stabilized the peripheral glia by preventing further Schwann-cell death. Additionally, our results showed that an axon-induced proximo-distal gradient of Schwann cell de-differentiation might represent a directional cue for regenerating axons to grow towards distal sites. This way, growth cones are induced to extend directionally along de-differentiated Schwann cells that are located exclusively distal to the site of damage (Allodi et al., 2012). It has previously been shown in mammals that de-differentiated Schwann cells promote axonal growth, and that Schwann cells re-differentiate after entering into contact with re-growing axons (Arthur-Farraj et al., 2012). We show here that Schwann cells in zebrafish re-differentiate and re-myelinate upon entering into contact with re-growing axons. Taken together, these results further support the strong conservation of the cellular processes underlying axon-glia interactions across vertebrates.

### How do sensory neurons respond to injury?

Time-course analyses of axons showed that, after severing, the distal segments degenerate, and that Schwann cells quickly clear many or most distal-axonal fragments by phagocytosis. Neurons remained viable in animals with severed axons, in which the severed axons began to regrow within 24 h post-damage. However, we found that axonal severing induces neuronal death in *ErbB2* mutant fish. The loss of ErbB2 blocked the migration and survival of most Schwann cells, suggesting that the peripheral glia might protect injured neurons from dying upon axonal damage. Notably, the survival of sensory spiral ganglion neurons requires ErbB signaling in the inner ear of mice (Stankovic et al., 2004). Because neuronal perikarya remain associated with Schwann cells in *ErbB2* mutant animals, a Schwann-cell-independent role of ErbB2 in neuronal survival remains a possibility. One way to test this idea is to induce post-injury activation or inhibition of ErbB2 in neurons.

### What is the identity of the directional cues for axonal regrowth?

We found that, after the physiological clearance of the degenerating distal axon fragments, the Schwann cells maintain endoneurial tubes, called bands of Büngner. It has been proposed that these tubes provide a permissive environment for axonal regeneration (Fawcett and Keynes, 1990; Feneley et al., 1991; Parrinello et al., 2010). However, they might also play an instructive role by inducing regrowing axons to extend growth cones in a directional manner. In rodents, nerve cuts trigger coordinated changes in behavior of fibroblasts and Schwann cells (Fawcett and Keynes, 1990; Feneley et al., 1991; Parrinello et al., 2010). This induces the collective migration of the Schwann cells towards the injury, guiding regrowing axons across the injury gap. Furthermore, if the collective migration of Schwann cells is impaired, axons take an abnormal path during regrowth (Fawcett and Keynes, 1990; Feneley et al., 1991; Parrinello et al., 2010). Using high-resolution intravital imaging, we complement and extend this model of glia-dependent axonal guidance during regeneration by showing that Schwann cells on both sides of the injury extend processes that rapidly bridge the gap. We also show that the directionality of this process is independent of the axons, suggesting that it emerges from the collective behavior of the Schwann cells. Axonal regeneration is slow before glial processes close the gap, but accelerates upon growth-cone contact with distal Schwann cells (Allodi et al., 2012; Bradke et al., 2012; Buettner et al., 1994). In addition, in the absence of Schwann cells, regenerating axons grow at slower rates and their growth cones stray and take random paths. These observations demonstrate similarities in multiple aspects of the role of the Schwann cells during axonal regeneration between mammals and fish. We do not know whether these similarities extend to fibroblasts, but live imaging in our zebrafish model of injury in combination with transgenic markers of fibroblasts might be able to address this question in the future. Importantly, our results strongly suggest that regenerating axons use haptotaxis with Schwann cells for directional growth. Therefore, Schwann cells have a powerful influence on the architecture of the repairing neural circuit by controlling the kinetics and directionality of axonal regrowth.

### Different modes of axonal repair

A protracted degeneration of distal axonal fragments could affect neuronal repair in two opposite ways: it might block axonal regrowth until the distal portion of the damaged fibers are completely cleared, but might also facilitate fast neuronal repair by axolemmal resealing (David et al., 1997; Spira et al., 1993). Sensory neurons are known to be under mechanical tension (Chada et al., 1997; Lafont and Prochiantz, 1994; Smith, 1988). In addition to trophic support (Soares et al., 2014), Schwann cells might control the mechanical forces acting on axons, which could bias the frequency of either regenerative pathway. In the future it will be important to characterize the physical environment of sensory neurons and the influence of the Schwann cells on the behavior of injured axons under different regimens of mechanical tension. To this end, the highly reproducible *in vivo* model of axonal injury that we present here and the anatomical simplicity and superficial localization of the zebrafish lateral line can be combined with genetically encoded sensors to visualize the mechanical properties of axons upon injury and repair in their natural context.

### Clinical implications

Peripheral neuropathies are sensory- and motor-neuron pathologies caused by genetic defects or stress (Nave, 2010; Cashman and Hoke, 2015). Comorbidity of traumatic and metabolic peripheral neuropathies is common in humans. Experimental and population-based studies have shown that lower-limb amputations due to neural dysfunction are prominent in individuals with diabetes (Lombardo et al., 2014). This invariably leads to a decrease in the quality of life of the affected individuals, and puts an enormous long-term burden on the healthcare systems. Notwithstanding recent progress, our knowledge about the influence of the glia on the degeneration and regeneration of the peripheral nervous system remains fragmentary (Faber and Merkies, 2015). Therefore, studies aimed at deepening our understanding of the dynamic interactions between neurons and Schwann cells in the natural context will be important to devise therapeutic strategies aimed at restoring sensory or motor function in humans suffering from traumatic injury. Collectively, our results show that denervation induces progressive destruction of the peripheral glia by increasing the apoptosis and inducing de-differentiation of Schwann cells. These negative effects of denervation are reversible, however, because re-innervation prevents further glial destruction. An important corollary of our results is the dominant influence of the Schwann cells on the onset and directionality of axonal regeneration. This has obvious clinical implications because strategies aimed at neural-circuit repair might benefit from preventing the death or disassembly of the peripheral glia during periods of denervation (Dedkov et al., 2002; Meier et al., 1999; Painter et al., 2014). We suggest that characterizing the axonal signals that control the differentiation status, entry into proliferation and survival of Schwann cells will provide multiple ‘druggable’ targets with therapeutic potential. One additional promising avenue is the screening of molecular libraries to identify drug leads. The genetic, anatomical and histopathological similarities of the peripheral glia among vertebrates suggest that the zebrafish model that we have developed is well suited for these approaches.

## MATERIALS AND METHODS

### Zebrafish strains and husbandry

Zebrafish were maintained under standardized conditions and experiments were conducted in embryos of undetermined sex in accordance with protocols approved by the Ethical Committee of Animal Experimentation of the Parc de Recerca Biomèdica de Barcelona, and the Institutional Animal Care and Use Committee of the Janelia Research Campus. Naturally spawned eggs were collected, cleaned and maintained in system water at 28.5°C at a density of 50 per 85-mm Petri dish. The Tg[gSAGFF202A] line was generated by random integration of a gene-trap construct (Asakawa et al., 2008). The Tg[Brn3c:mEGFP], Tg[SILL1] and Tg[UAS:EGFP] transgenic lines have been described previously (Faucherre and López-Schier, 2011, 2014; Faucherre et al., 2009; Xiao et al., 2005). The *ErbB2^st61^* mutant allele was obtained from W. Talbot (Stanford University, CA).

### Selection of mutants

Wild-type animals, and homozygous and trans-heterozygous mutants for *ErbB2^st61^*, were incubated in DiASP. Homozygous mutants displayed extra neuromasts, whereas wild-type and heterozygous larvae displayed only seven to nine neuromasts on one side of their trunk. Heterozygous Tg[gSAGFF202A] was genotyped using the primers: Forward: 5′-GCTCAAGTGCTCCAAAGAAA-3′; Reverse: 5′-ATCAGCAGGCAGCATGTCC-3′. Heterozygous and homozygous Tg[gSAGFF202A;SILL:mCherry] were screened with a stereomicroscope under ultraviolet light. Lateral line nerves of heterozygous larvae developed along the horizontal myoseptum, whereas those of homozygous mutants were more ventral.

### DNA constructs

The UAS:H2A-mTurquoise, hsp70:EGFP-SILL (SILL:EGFP) and UAS:EGFP-UtrCH constructs were generated using the Tol2 kit. Entry vectors were generated as described in the Invitrogen Multisite Gateway manual. PCRs were performed using primers to add *att* sites onto the end of DNA fragments, using Platinum Pfx (Invitrogen). The pEntry vectors containing the UAS sequence, *hsp70* minimal promoter, *EGFP* and polyA are from the Tol2 kit, and the pEntry vector containing the SILL enhancer has been previously generated by our laboratory. To generate the middle entry clone containing H2A-mTurquoise cDNA (using pDONR 221), the forward PCR primer containing an *attB1* site and the reverse primer containing an *attB2* site were used: Forward: 5′-GGGGACAAGTTTGTACAAAAA-AGCAGGCTGCCACCATGGTGAGCAAGGGCGA-3′; Reverse: 5′-GGGGACCACTTTGTACAAGAAAGCTGGGTTTATTTGCCTTTGGCCTTGTG-3′. To generate the middle entry clone containing EGFP-UtrCH, the forward primer was the same as that for H2A-mTurquoise, and combined with: Reverse: 5′-GGGGACCACTTTGTACAAGAAAGCTGGGTTTAGTCTATGGTGACTTGCTG-3′.

### Generation of transgenic zebrafish

To generate the Tg[UAS:H2A-mTurquoise] stable transgenic line, 20 pg of the Tol2-expression clone and 20 pg of the transposase synthetic RNA were simultaneously injected into one-cell-stage wild-type eggs. The resulting embryos were raised to adulthood and out-crossed to Tg[gSAGFF202A;SILL:mCherry] for visual inspection for presence of the transgene.

### Mosaic gene expression

To transiently and stochastically express transgenes in neurons, 25-30 pg of plasmids containing the SILL enhancer were injected into embryos at the one- or two-cell stage.

### BrdU incorporation

To quantify Schwann-cell proliferation by means of DNA synthesis, transgenic Tg[gSAGFF202A;UAS:EGFP;SILL:mCherry] larvae at 3 dpf were incubated immediately after axonal severing in medium containing 5-bromo-2′-deoxyuridine (10 mM) for 24 h. Larvae were fixed at 24 hpt and immunolabeled.

### Antibody staining

For immunohistochemistry, samples were fixed overnight at 4°C in a solution of 4% paraformaldehyde in phosphate-buffered saline (PBS) solution containing 1% Tween-20 (PBST). After fixation, the samples were washed in the same solution without fixative and blocked at room temperature with 10% bovine serum albumin. Primary- and secondary-antibody incubations were conducted overnight at 4°C in PBS with 0.2% Tween-20. Primary antibodies were used at the following dilutions: mouse monoclonal antibody 6D2 (obtained from G. Jeserich, Universität Osnabrück), 1/5; rat anti-claudin-k, 1:1000 (obtained from T. Becker, University of Edinburgh). Texas-Red-labeled donkey anti-mouse and -rat immunoglobin secondary antibodies (Jackson Labs) were used at a dilution of 1:200. For BrdU immunohistochemistry, zebrafish larvae were fixed as above and subsequently rinsed in PBST and 2 M HCl before being blocked in 10% goat serum. Samples were incubated in rat anti-BrdU primary antibody (1:100; Abcam) followed by goat anti-rat Alexa-Fluor-568 secondary antibody (1:300). Images were obtained using a confocal microscope (Sheppard and Wilson, 1981) (LSM 510; Carl Zeiss).

### Detection of cell death

Apoptosis was assessed by the terminal deoxynucleotidyl transferase dUTP nick end labeling (TUNEL) assay. Samples were fixed with 4% paraformaldehyde overnight at 4°C and dehydrated with methanol at −20°C. After gradual rehydration, the larvae were permeabilized with 20 μg/ml proteinase K for 20 min followed by 4% paraformaldehyde, and incubated with 90 μl labeling solution plus 10 μl enzyme solution (In Situ Cell Death Detection Kit, Fluorescein, Roche) at 37°C for 3 h in darkness. They were washed three times with PBST for 15 min each and the images were examined by confocal microscopy.

### Live imaging

For live imaging, larvae were anesthetized with a 610 μM solution of the anesthetic 3-aminobenzoic acid ethyl ester (MS-222) and mounted onto a glass-bottom 3-cm Petri dish (MatTek) and covered with 1% low-melting-point agarose with diluted anesthetic. Images were acquired with an inverted laser-scanning confocal microscope, or an inverted spinning-disc confocal microscope (Visitron), with a 40× air or 63× water-immersion objective. *z*-stacks of Schwann cells and axons consisted of 1-μm-spaced images, which were analyzed with ImageJ software.

### Lattice light-sheet microscopy

For live imaging by lattice light-sheet microscopy, the specimens were anesthetized with MS-222. Samples were then suspended in molten 1% low-melting-point agarose containing diluted MS-222 and mounted on #1 5-mm round coverslips (CS-5R, Warner Instruments). Sample and coverslip were stabilized in a custom-made stainless-steel holder and attached to piezo stages (Physik Instrumente). Imaging was conducted at room temperature (21°C) in PBS. Images were acquired via 1.1 NA 25× water-dipping objective (Nikon). Bessel-beam plane illumination microscopy has been previously published (Gao et al., 2014; Planchon et al., 2011). Briefly, for lattice light-sheet imaging, a collimated 488-nm or 561-nm laser light was passed through a pair of cylindrical lenses to illuminate a stripe across the width of a ferroelectric spatial light modulator (SLM, Forth Dimension Displays). The optical path following the SLM creates a demagnified image of the pattern at the focal plane of a custom-made water-dipping excitation objective (Special Optics, 0.65 NA). The lattice pattern was dithered along the *x*-axis and swept through the *z*-axis to create 3D volumes. Deconvolution was performed using Richardson-Lucy iterations and movies were made using the Amira software (FEI).

### Quantification of Schwann cells

To quantify Schwann cells, images of a 1-mm-long segment were taken at the distal part of the injury and followed for 5 days. Schwann-cell nuclei were counted using H2A-mTurquoise expression, EGFP and BrdU.

### Laser-mediated axon severing and cell ablation

For axonal severing or cell ablation we used the iLasPulse laser system (Roper Scientific SAS) mounted on a Zeiss Axio Observer inverted microscope equipped with a 63× water objective lens. Zebrafish larvae were anesthetized, mounted on a glass-bottom dish and covered with methylcellulose. A train of laser pulses was repeatedly applied to the posterior lateralis ganglion or lateral axons and Schwann cells until all red or green fluorescence disappeared. Samples were assessed again for the presence of mCherry or EGFP in the target region 1 h after axonal severing or cell ablation. Total ablation occurred in samples with no red-fluorescent or green-fluorescent signal in the target region. Laser microsurgery was conducted in over 100 animals, resulting in more than 90% survival.

## Supplementary Material

Supplementary Material
