# Supplementary material for: High-resolution live imaging reveals axon-glia interactions during peripheral nerve injury and repair in zebrafish
Source: Dis Model Mech. 2015 Jun 1;8(6):553–64. doi: 10.1242/dmm.018184 (PMC4457030; doi:10.1242/dmm.018184)
Supplement: Supplementary Material [file supp_8_6_553__index.html]

High-resolution live imaging reveals axon-glia interactions during peripheral nerve injury and repair in zebrafish — Supplementary Material 

# High-resolution live imaging reveals axon-glia interactions during peripheral nerve injury and repair in zebrafish

## DMM018184 Supplementary Material

**Files in this Data Supplement:**

- **Supplementary Material**
