# Supplementary material for: High-resolution live imaging reveals axon-glia interactions during peripheral nerve injury and repair in zebrafish
Source: Dis Model Mech. 2015 Jun 1;8(6):553–64. doi: 10.1242/dmm.018184 (PMC4457030; doi:10.1242/dmm.018184)
Supplement: Supplementary Material [file supp_8.6.553_DMM018184.pdf]

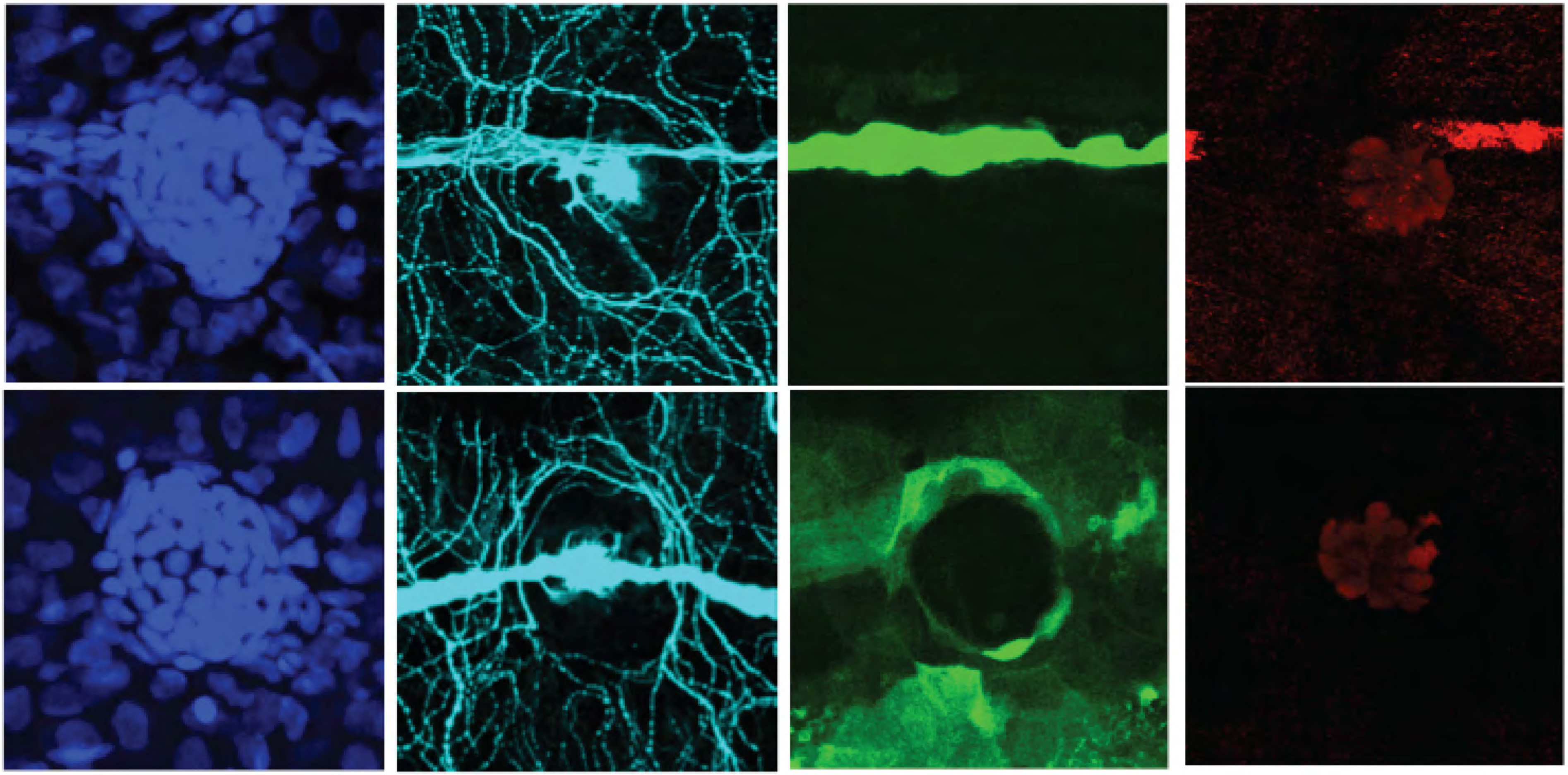

Supplementary Figure 1. This series of images further show that Schwann cells are absent in along the trunk of the fish with a homozygous Tg[gSAGFF-202A] insertion. While EGFP (green) in Tg[gSAGFF-202A;UAS:EGFP] heterozygous fish at 96 hpf is expressed in Schwann cells surrounding the lateral-line afferent axons labeled with and antibody to acetylated tubulin (turquoise), the EGFP signal is absent from the perineurium, and appears upregulated in neuromasts in Tg[gSAGFF- 202A;UAS:EGFP] homozygous fish. The lateral line contains hair cells, which were labeled with an antibody to S100 (red). Cellular nuclei were labeled with DAPI (blue).

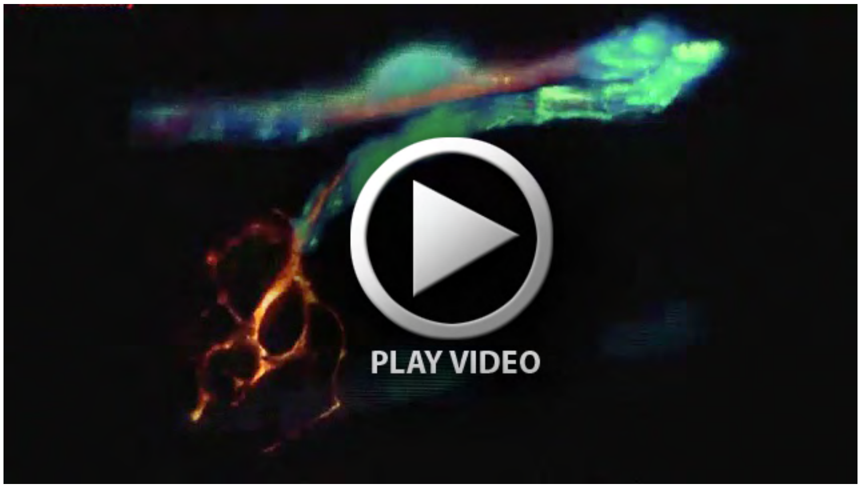

Supplementary Movie 1. Axial rotation of a volumetric representation of Schwann cells (green) and axons (red) at high resolution obtained by lattice light sheet imaging in Tg[gSAGFF-202A;UAS:EGFP; SILL:mCherry] fish at 5 dpf.

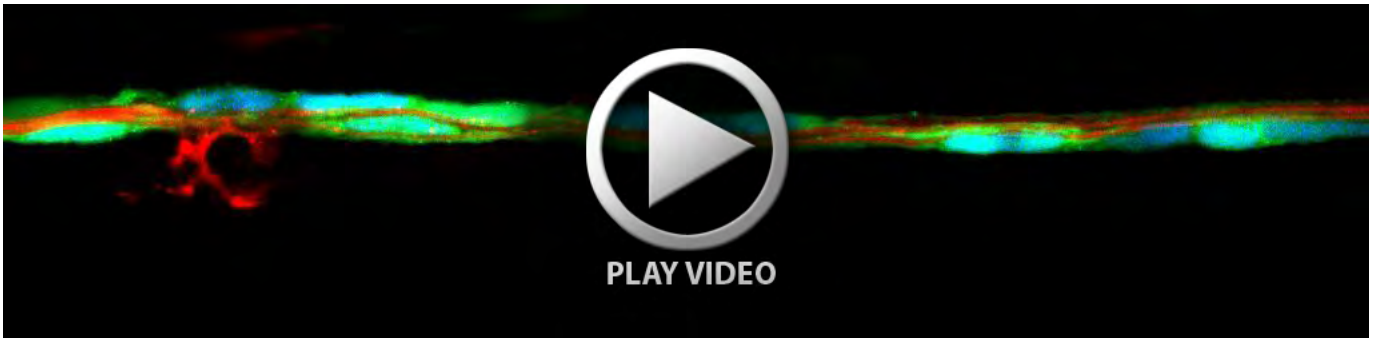

Supplementary Movie 2. This time-lapse sequence shows the dynamics of Schwann cells and axons at 3 dpf Tg[gSAGFF-202A;UAS:EGFP; UAS:H2A-mTurquoise; SILL:mCherry] fish after axonal severing. This sequence clearly shows Schwann cells experience cell death and can actively divide when there is no intact axon. The movie starts immediately after axonal severing and takes images every 7min for 18h 33min at 1 frame per second (fps).

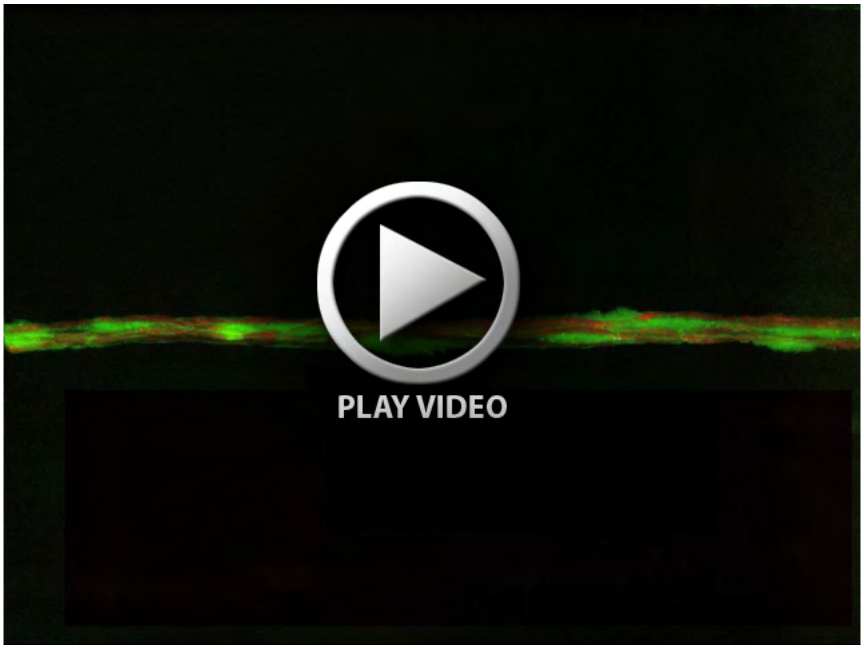

Supplementary Movie 3. Time-lapse sequence showing the dynamics of Schwann cells and axons at 5 dpf Tg[gSAGFF-202A;UAS:EGFP; SILL:mCherry] fish after axonal severing leaving glial gap of 20 um. The movie starts immediately after axonal severing and takes images every 15 min for 20 h at 2 fps.

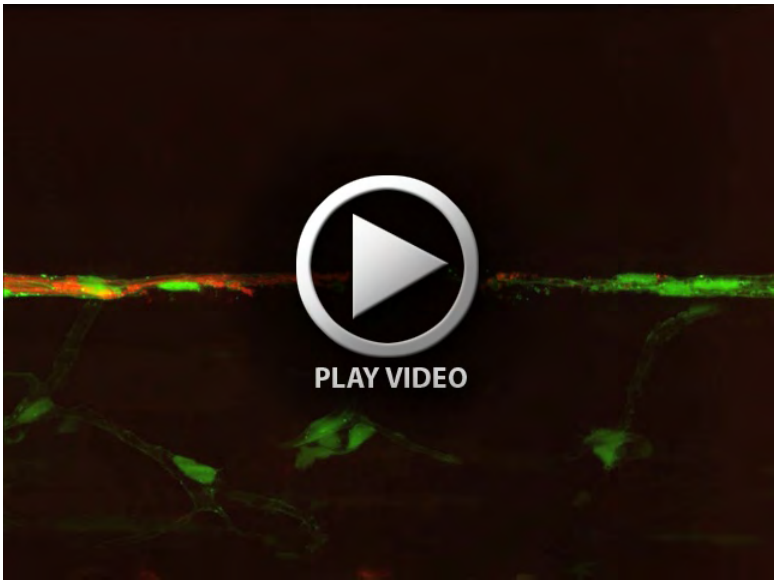

Supplementary Movie 4. This time-lapse sequence shows the dynamics of Schwann cells and axons at 6 dpf Tg[gSAGFF-202A;UAS:EGFP; SILL:mCherry] fish after axonal severing leaving glial gap of 50 um. The movie starts 4 hours after axonal severing and takes images every 8 min for 14 h at 2 fps.

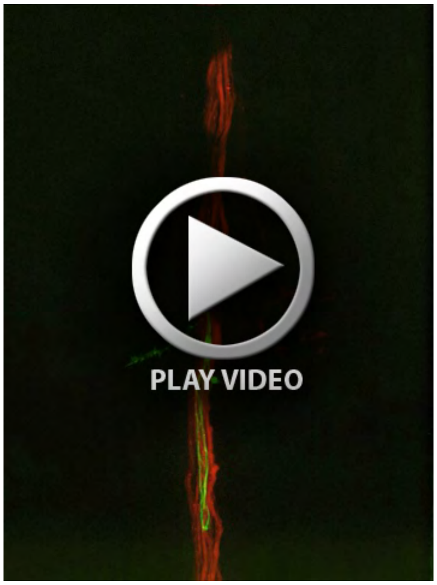

Supplementary Movie 5. This time-lapse sequence shows the dynamics of individual Schwann cell and axons in 5 dpf Tg[gSAGFF-202A;SILL:mCherry] fish injected with UtrCH-EGFP DNA before axonal severing. The images were taken every 3 min for 1 h at 2 fps

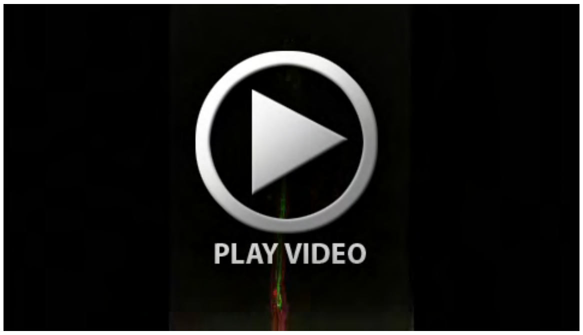

Supplementary Movie 6. This time-lapse sequence shows the dynamics of individual Schwann cell and axons at 5 dpf Tg[gSAGFF-202A; SILL:mCherry] injected with UtrCH-EGFP DNA immediately after axonal severing. The images take every 3 min for 13 h at 2 fps.
